# Subchordal Cyst Containing Non-degraded Hyaluronic Acid: A Rare Complication of Injection Laryngoplasty

**DOI:** 10.7759/cureus.27801

**Published:** 2022-08-08

**Authors:** Nur Asyiqin Kamarudin, Norazila Abdul Rahim, Mawaddah Azman, Marina Mat Baki

**Affiliations:** 1 Department of Otorhinolaryngology and Head and Neck Surgery, Faculty of Medicine, Universiti Kebangsaan Malaysia Medical Centre, Kuala Lumpur, MYS; 2 Department of Otorhinolaryngology, Faculty of Medicine, Universiti Teknologi MARA (UiTM), Puncak Alam, MYS

**Keywords:** marsupialization, subchordal cyst, delayed complication, hyaluronic acid, injection laryngoplasty

## Abstract

Injection laryngoplasty (IL) with hyaluronic acid (HA) for the treatment of glottic insufficiency has been reported as a safe procedure with low complication rates. The side effects usually present as acute dyspnea, dysphonia, or dysphagia due to local edema, hematoma, or hypersensitivity reactions. These complications commonly resolve with minimal intervention. In the present case, we present an asymptomatic case of a subchordal cyst (SC) in a 30-year-old female after office-based injection laryngoplasty using hyaluronic acid. To the best of our knowledge, this is the first reported case of a subchordal cyst following injection laryngoplasty.

## Introduction

Hyaluronic acid (HA) is a linear polysaccharide of glycosaminoglycan present in the lamina propria of the vocal fold extracellular matrix [1]. Its biocompatible, biodegradable, and viscoelastic properties [2] render HA suitable for use as a temporary injectate in injection laryngoplasty (IL). Unmodified HA has a half-life of approximately 24 hours [3]. Thus, for use as a dermal filler, HA is modified by crosslinking or conjugation to reduce its degradation rate [2]. IL using HA is safe, with a complication rate of just 3.8%-4.7%, mainly due to local hypersensitivity and inflammation [1]. In a systematic review of 14 studies, only two adverse effects were described: vocal fold hematoma and aryepiglottic fold edema [1]. Here, we present a case of subchordal cyst (SC) formation following IL using HA.

## Case presentation

**Figure 1:**
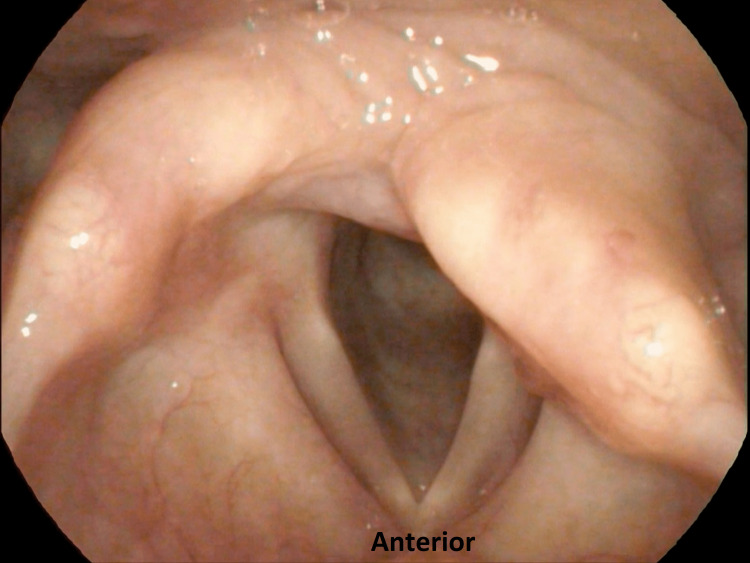
Video laryngoscopy view of laryngeal inlet prior to IL that showed bowing of the left vocal fold in the lateral position

A 30-year-old female underwent hemithyroidectomy for a left multinodular goiter where the recurrent laryngeal nerve was preserved. However, she experienced hoarseness, voice fatigue, and dysphagia with liquids including concern for aspiration with drinking fluids following the surgery for one month prior to referral to our center. Her voice-related quality of life as measured using the Malay version of the Voice Handicap Index-10 (mVHI-10) [4] was 34/40, indicating severe voice-related handicap. The score for Eating Assessment Tool (EAT-10) was abnormal, which was 13. The perceptual evaluation of her voice using the overall grade of dysphonia, roughness, breathiness, asthenia, and strain (GRBAS) scale [5] showed a severe deviation of voice with the main component of breathiness and asthenia (G3R0B3A3S1). Furthermore, her maximum phonation time was shortened to three seconds. Video laryngoscopy revealed a bowed and immobile left vocal fold in the lateral position with a large phonatory gap (Figure 1) (Video 1).

**Video 1:** Video laryngoscopy revealing the immobile left vocal cord with a large phonatory gap and anteromedial prolapse of the right arytenoid upon phonation

Videostroboscopy revealed an asymmetrical mucosal wave with a decrease in amplitude on the left vocal fold (Video 2).

**Video 2:** Videostroboscopy showing that the left vocal fold was in the left lateral position with asymmetrical mucosal wave and decreased amplitude

**Figure 2:**
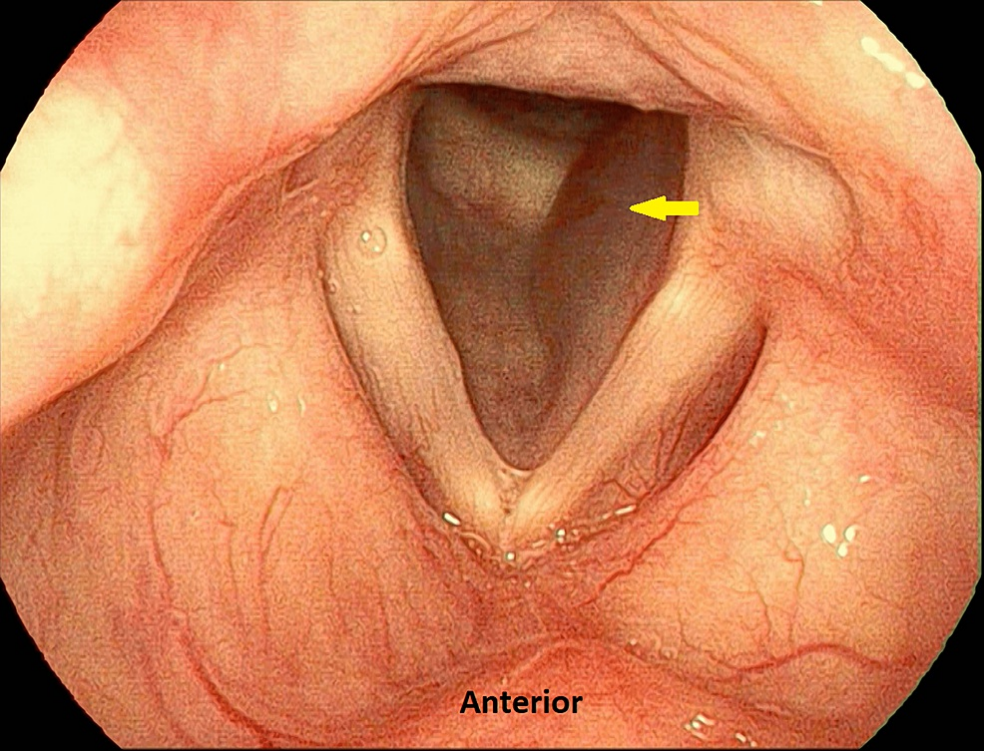
Smooth translucent swelling (arrow) in the left subglottic space three months post-IL, suggestive of a subchordal cyst

IL was performed in a clinical setting under local anesthesia. HA (Juvéderm Ultra XC, Allergan, USA) was injected into the left paraglottic space lateral to the left vocal fold via the trans-thyrohyoid approach using a double-bend needle [6]. It was confirmed that the injectates has not passed the expiry date prior to the procedure. The follow-up appointment was delayed due to COVID-19-related restrictions. At three months post-injection, her voice returned to normal (mVHI-10 score of 2/40). Video laryngoscopy revealed return of left vocal fold mobility. However, there was a smooth translucent swelling seen in the left subglottic region with no airway obstruction (Figure 2).

**Figure 3:**
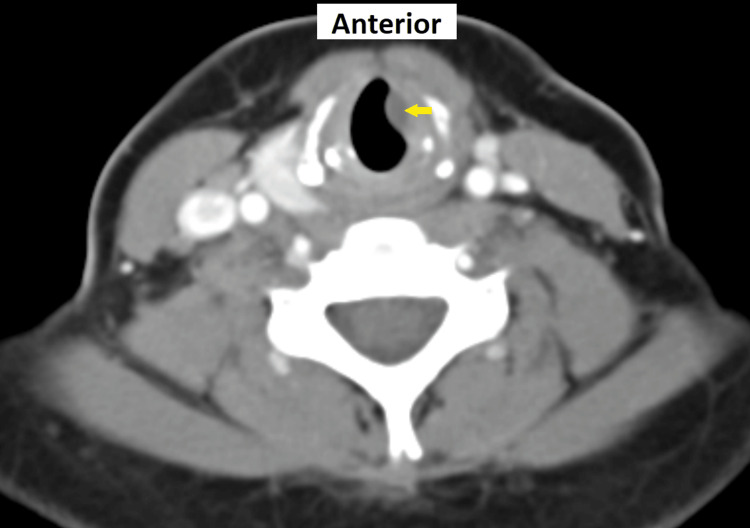
CECT scan of the neck showing a well-defined hypodense lesion (arrow) at the left subglottic region representing subchordal cyst

The swelling remained the same for 20 months post-IL. Contrast-enhanced computed tomography (CECT) scan of the neck showed a non-enhancing, well-defined, hypodense lesion arising in the left side of the subglottic region (0.8 × 0.4 × 0.8 cm) (Figure 3).

A diagnosis of acquired subchordal cyst secondary to IL was made.

**Figure 4:**
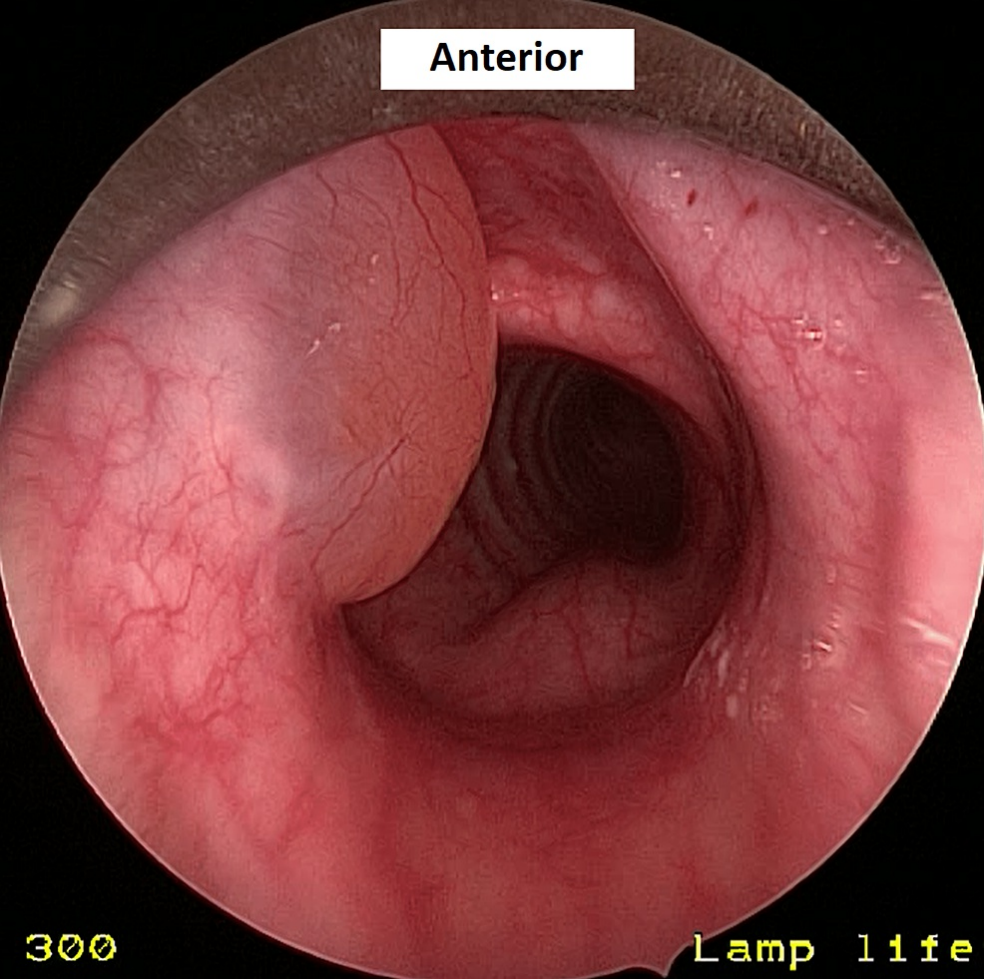
Left subglottic cyst arising from the left vocal cord’s inferior surface upon direct laryngoscopy; the cyst remains the same in size and appearance after 20 months since diagnosis

The patient underwent marsupialization of the cyst under general anesthesia with tubeless ventilation in which the larynx was visualized and suspended using a rigid subglottic laryngoscope. The cyst was found to be located in the subglottic region adjacent to the inferior surface of the left vocal fold (Figure 4).

**Figure 5:**
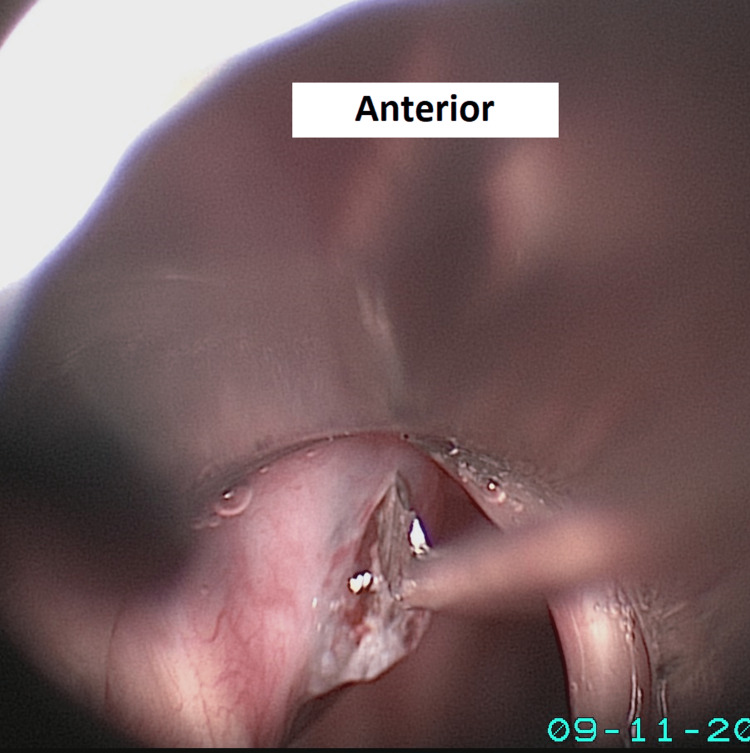
Gel-like material extruded upon marsupialization

Upon marsupialization, gel-like material resembling HA was observed (Figure 5) (Video 3).

**Video 3:** Marsupialization of the subchordal cyst containing hyaluronic acid

Histopathological evaluation of the SC wall showed amorphous material positive for Alcian blue and negative for periodic acid-Schiff stain with surrounding foreign body reaction suggesting exogenous HA.

The patient was reviewed in the clinic two weeks post-marsupialization. Her voice was normal, and videostroboscopy revealed normal vocal folds with a symmetrical mucosal wave.

## Discussion

HA can bind to 1,000 times its weight in water. Following injection into the tissue, it will absorb water and slowly expand within 24 hours [3]. This delayed swelling may explain why the SC was not observed earlier during the procedure. Initially, we postulated that the SC was formed due to the mechanical obstruction of the ductal systems draining the seromucinous glands of the vocal fold, which lead to a collection of mucus-filled cavities lined by the epithelium. However, the intraoperative findings and histopathology results clearly indicated the cyst to contain exogenous HA.

In vivo, 70% of HA is catabolized systemically, where it is transported via the lymphatic or blood vessels to the lymph node or liver, respectively. It is then internalized and catabolized by endothelial cells. The remaining 30% is degraded locally by hyaluronidase or oxidative damage by reactive oxygen species [2]. Thus, mechanical compression of the lymphatic and vascular supply by HA may explain why it was not degraded in our patient. Furthermore, the utilized HA (Juvéderm Ultra XC) is more resistant to hyaluronidase degradation when compared with the Restylane family [7]. HA fillers differ in their cohesivity, viscosity, and response to hyaluronidase. This variation depends on the extent of the crosslinking, concentration, particulate size, and mono- or biphasic status. The monophasic form (cohesive gel without particles) is more resistant to hyaluronidase because biphasic filler (particles suspended in gel) has a greater surface area for enzymatic attack. Both factors, being monophasic with higher crosslinking and higher HA concentrations may explain why Juvéderm is more resistant to resorption and degradation [7].

In our setting, IL was done percutaneously under flexible endoscopic laryngoscopy guidance. The HA is aimed to be injected into the paraglottic space. However, even under laryngoscopy guidance, the tip of the needle still cannot be seen clearly, resulting in the risk of the material being injected either too superficial or too deep. Thus, another possibility for the development of SC in this case was that the tip of the needle went beyond the conus elasticus and was injected submucosally at the inferior surface of the vocal fold. To overcome this problem, Wang et al. have suggested performing IL under laryngeal electromyography (LEMG) guided to ensure accurate infiltration of the material at the thyroarytenoid and lateral cricoarytenoid complex [1].

HA has been known to be biodegradable, where it typically lasts for 4-6 months, with beneficial outcomes seen up to 12 months post-IL [1]. To the best of our knowledge, this is the first case described in the literature of SC containing non-degradable HA secondary to IL.

## Conclusions

SC is a rare complication following IL using HA. Routine follow-up is crucial given the possibility of delayed complications following IL. This report suggests that HA may persist for at least 20 months without degradation following IL. Management of the SC containing non-degraded HA with marsupialization showed a good outcome with complete resolution and no adverse sequelae.
